# A Ca^2+^-binding motif underlies the unusual properties of certain photosynthetic bacterial core light-harvesting complexes

**DOI:** 10.1016/j.jbc.2022.101967

**Published:** 2022-04-20

**Authors:** Kazutoshi Tani, Kazumi Kobayashi, Naoki Hosogi, Xuan-Cheng Ji, Sakiko Nagashima, Kenji V.P. Nagashima, Airi Izumida, Kazuhito Inoue, Yusuke Tsukatani, Ryo Kanno, Malgorzata Hall, Long-Jiang Yu, Isamu Ishikawa, Yoshihiro Okura, Michael T. Madigan, Akira Mizoguchi, Bruno M. Humbel, Yukihiro Kimura, Zheng-Yu Wang-Otomo

**Affiliations:** 1 Graduate School of Medicine, Mie University, Tsu, Japan; 2 EM Business Unit, JEOL Ltd 3-1-2 Musashino, Akishima, Tokyo, Japan; 3 Faculty of Science, Ibaraki University, Mito, Japan; 4 Research Institute for Integrated Science, Kanagawa University, Hiratsuka, Kanagawa, Japan; 5 Department of Biological Sciences, Faculty of Science, Kanagawa University, Hiratsuka, Kanagawa, Japan; 6 Institute for Extra-cutting-edge Science and Technology Avant-garde Research (X-star), Japan Agency for Marine–Earth Science and Technology (JAMSTEC), Kanagawa, Japan; 7 Imaging Section, Research Support Division, Okinawa Institute of Science and Technology Graduate University (OIST), Kunigami-gun, Okinawa, Japan; 8 Photosynthesis Research Center, Key Laboratory of Photobiology, Institute of Botany, Chinese Academy of Sciences, Beijing, China; 9 School of Biological Sciences, Department of Microbiology, Southern Illinois University, Carbondale, Illinois, USA; 10 Department of Agrobioscience, Graduate School of Agriculture, Kobe University, Nada, Kobe, Japan

**Keywords:** bioenergetics, biophysics, calcium-binding protein, cryo-electron microscopy, light-harvesting complex (antenna complex), photosynthesis, photosynthetic pigment, BChl, bacteriochlorophyll, LH1, light-harvesting complex, RC, reaction center

## Abstract

The mildly thermophilic purple phototrophic bacterium *Allochromatium tepidum* provides a unique model for investigating various intermediate phenotypes observed between those of thermophilic and mesophilic counterparts. The core light-harvesting (LH1) complex from *A. tepidum* exhibits an absorption maximum at 890 nm and mildly enhanced thermostability, both of which are Ca^2+^-dependent. However, it is unknown what structural determinants might contribute to these properties. Here, we present a cryo-EM structure of the reaction center–associated LH1 complex at 2.81 Å resolution, in which we identify multiple pigment-binding α- and β-polypeptides within an LH1 ring. Of the 16 α-polypeptides, we show that six (α1) bind Ca^2+^ along with β1- or β3-polypeptides to form the Ca^2+^-binding sites. This structure differs from that of fully Ca^2+^-bound LH1 from *Thermochromatium tepidum*, enabling determination of the minimum structural requirements for Ca^2+^-binding. We also identified three amino acids (Trp44, Asp47, and Ile49) in the C-terminal region of the *A. tepidum* α1-polypeptide that ligate each Ca ion, forming a Ca^2+^-binding WxxDxI motif that is conserved in all Ca^2+^-bound LH1 α-polypeptides from other species with reported structures. The partial Ca^2+^-bound structure further explains the unusual phenotypic properties observed for this bacterium in terms of its Ca^2+^-requirements for thermostability, spectroscopy, and phototrophic growth, and supports the hypothesis that *A. tepidum* may represent a “transitional” species between mesophilic and thermophilic purple sulfur bacteria. The characteristic arrangement of multiple αβ-polypeptides also suggests a mechanism of molecular recognition in the expression and/or assembly of the LH1 complex that could be regulated through interactions with reaction center subunits.

Bacterial photosynthetic antennae have evolved diverse strategies to optimize their spectral and thermodynamic properties for adaptation to changing environments. For the core light-harvesting complex (LH1) of purple phototrophic bacteria, the first thoroughly investigated case was that of the thermophilic sulfur bacterium, *Thermochromatium tepidum*, a phototroph isolated from a hot spring microbial mat in Yellowstone National Park (USA) and capable of growth up to 57 °C (1). This bacterium incorporates calcium ions into its LH1 complex, resulting in an enhanced thermostability and a red-shifted absorption maximum (Q_y_ transition) at 915 nm (2, 3, 4, 5, 6). Subsequent crystallographic structure of the *T. tepidum* LH1 in a reaction center (RC)-associated form identified 16 Ca^2+^-binding sites in the LH1 complex (7), each Ca^2+^ being bound by a pair of αβ-polypeptides and two water molecules (8). This Ca^2+^-binding network tightly locks the LH1 ring and contributes to both its thermostability and Q_y_ redshift (9, 10, 11).

The dual role of Ca^2+^ in affecting LH1 properties was also demonstrated in a second case where the mesophilic purple sulfur bacterium *Thiorhodovibrio* (*Trv.*) strain 970 was shown to integrate Ca^2+^ into its LH1 complex (12, 13), triggering an even greater redshift of its LH1 to 960 nm, the most red-shifted Q_y_ of all bacteriochlorophyll (BChl) *a*-containing species. A recent cryo-EM structure of the *Trv.* strain 970 LH1–RC complex revealed that 16 Ca^2+^ are present in the LH1 C-terminal domain and a Ca^2+^-facilitated hydrogen-bonding network forms the structural basis of the unique LH1 redshift (14). By contrast, a completely different mechanism contributes to the thermostability of the LH1 of a BChl *b*-containing thermophilic purple nonsulfur bacterium *Blastochloris tepida* (15, 16). This organism synthesizes more carotenoids with longer conjugations relative to its mesophilic counterpart. However, although conferring thermostability, the altered carotenoid composition had no effect on *B. tepida* LH1-Q_y_ absorption (16).

There also exist intermediate cases for the LH1 complexes that exhibit various properties in between those of thermophilic and mesophilic purple sulfur bacteria. An example is the LH1–RC isolated from a mildly thermophilic purple sulfur bacterium *Allochromatium tepidum* (17). This organism grows optimally near 45 °C, a temperature between the optima of the thermophilic *T. tepidum* (∼50 °C) and mesophilic *Allochromatium vinosum* (∼30 °C). The LH1–RC complex purified from *A. tepidum* shows an LH1-Q_y_ absorption at 890 nm (Fig. S1) and a mildly enhanced thermostability (18). Both of the properties are Ca^2+^-dependent and intermediate between those of *T. tepidum* and *A. vinosum*. By biochemical analysis (18), approximately five Ca ions were detected per *A. tepidum* LH1–RC, significantly fewer than that in the *T. tepidum* LH1–RC. This observation motivated us to examine the *A. tepidum* LH1–RC in more detail, and here, we present its cryo-EM structure. The structure identified six Ca ions in the LH1 complex that are specifically bound to a specific motif in certain α-polypeptides and offers insights into spectroscopic and thermodynamic behaviors of the *A. tepidum* LH1. Moreover, the detailed density map of the *A. tepidum* LH1–RC complex reveals the structural arrangement of multiple forms of αβ-polypeptides in the LH1 ring that explain the many intermediate properties of this complex compared with those of *T. tepidum* and *A. vinosum*.

## Results

### Structural overview

**Figure 1:**
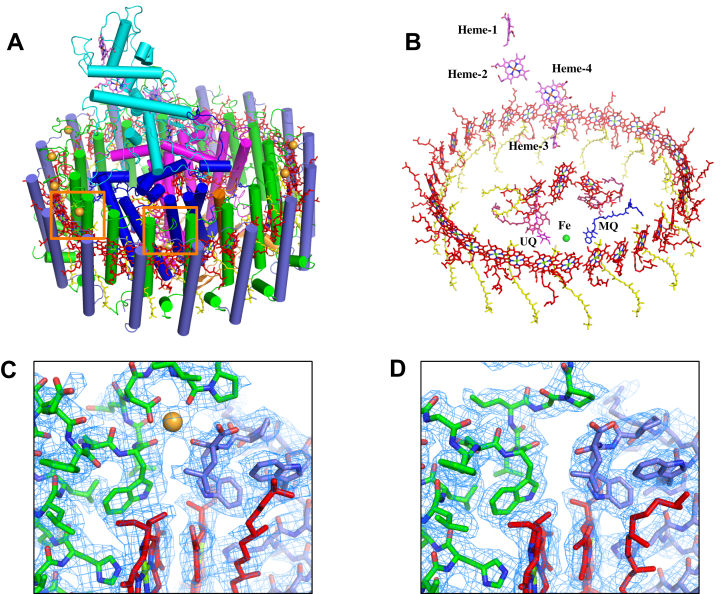
**Overall structure and cofactor arrangement of the *Allochromatium tepidum* LH1–RC complex.***A*, tilted view of the LH1–RC with the periplasmic C-subunit (*cyan*) above. *B*, tilted view of the cofactor arrangement with the periplasm above and the cytoplasm below. *C*, a typical Ca^2+^-binding site (marked in (*A*) by the *orange rectangle* on the *left-hand side*) with the density map around the C-termini of LH1-α1 and LH1-β3 polypeptides. *D*, a typical Ca^2+^-free site (marked in (*A*) by the *orange rectangle* on the *right-hand side*) with the density map around the C-termini of LH1-α3 and LH1-β1 polypeptides. Color scheme: LH1-α, *green cylinders*; LH1-β, *slate blue cylinders*; L-subunit, *magenta cylinder*; M-subunit, *blue cylinder*; C-subunit, *cyan cylinder*; Ca^2+^, *orange ball*; BChl *A*, *red sticks*; spirilloxanthin, *yellow sticks*; MQ, *blue sticks*; UQ, *magenta sticks*. Phospholipids and detergents are omitted for clarity. The density maps are shown at a contour level of 4.0σ. BChl, bacteriochlorophyll; LH1, light-harvesting complex; RC, reaction center.

**Figure 2:**
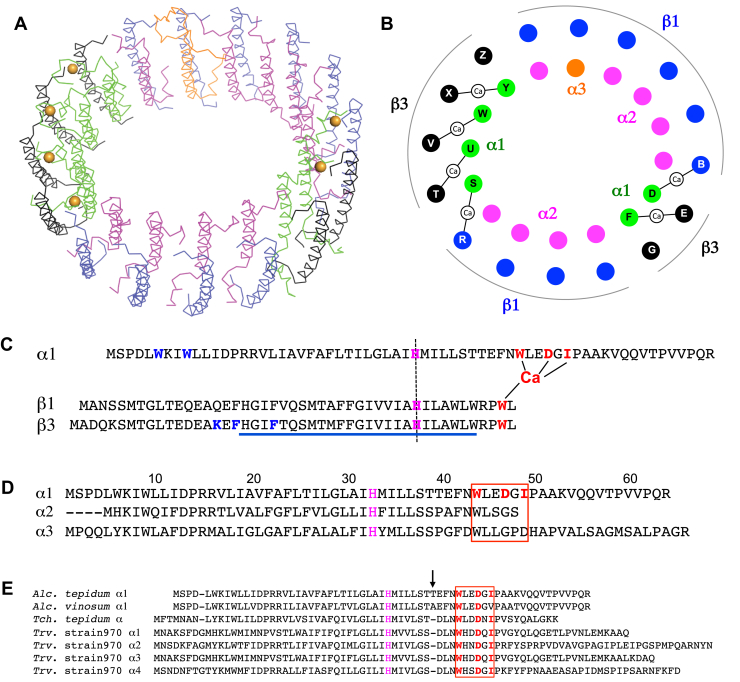
**Ca**^**2+**^**-binding motif and arrangement of the *Allochromatium tepidum* LH1 multiple polypeptides.***A*, tilted view of the LH1–RC from the periplasmic side of the membrane. Color scheme: α1, *green*; α2, *magenta*; α3, *orange*; β1, *blue*; β3, *black*; Ca^2+^, *orange ball*. *B*, illustration of the arrangement of the LH1 polypeptides. Letters in the *colored circles* denote chain IDs. Color scheme as in (*A*). *C*, sequence alignment showing the relative positions of the Ca^2+^- and BChl *a*-binding sites. The Ca^2+^-bound αβ-polypeptides are aligned relative to the BChl *a*-coordinating histidine residues (*magenta* letters with *vertical dashed line*). The Ca^2+^-ligating residues are shown in *red letters*. Key residues interacting in the N-terminal regions of α1- and β3-polypeptides are shown in *blue letters*. The underlined region represents a predicted membrane-spanning domain. *D*, sequence alignment of the α1-, α2-, and α3-polypeptides relative to the BChl *a*-coordinating histidine residues showing that the specific Ca^2+^-binding motif (WxxDxI) is only present in the α1-polypeptide. *E*, sequence alignment of the *A. tepidum* α1-polypeptide with those of *A. vinosum*, *T. tepidum* and *Trv*. strain 970 relative to the BChl *a*-coordinating histidine residues. The Ca^2+^-binding motif (WxxDxI) of *T. tepidum* and *Trv*. strain 970 (*red box*) is not present in the *A. vinosum* α1-polypeptide. An insertion in the *A. tepidum* and *A. vinosum* α1-polypeptides is indicated by an *arrow*. BChl, bacteriochlorophyll; LH1, light-harvesting complex; RC, reaction center.

The cryo-EM structure of *A. tepidum* LH1–RC was determined at 2.81 Å resolution (Table S1 and Figs. S2 and S3), and its overall structure reveals some features shared with *T. tepidum* (8) and *Trv.* strain 970 LH1–RCs (14). The *A. tepidum* LH1 forms a closed, slightly elliptical ring structure composed of 16 pairs of helical α(inner)β(outer)-polypeptides, 32 BChls *a*, and 16 all-*trans* spirilloxanthins that are uniformly distributed around the RC (Figs. 1 and S4). Multiple genes encoding the LH1 α- and β-polypeptides were confirmed in the genome (Fig. S5). Three forms of α-polypeptide and two forms of β-polypeptide were identified in the density maps which are consistent with biochemical analyses (Fig. 2, S4 and S5). Six Ca ions were detected in the density map that are specifically bound to the LH1 α1- and β-polypeptides (Figs. 1, *C* and *D* and 2). The Ca^2+^ stoichiometry is consistent with that determined by inductively coupled plasma atomic emission spectroscopy (18). The RC has a tetraheme-cytochrome subunit and contains four BChls *a*, two bacteriopheophytins *a*, one 15-*cis*-spirilloxanthin, a menaquinone (MQ)-8 at the Q_A_ site, and a ubiquinone (UQ)-8 at the Q_B_ site. The RC fits the shape of the inner LH1 α-ring with the L- and M-subunits in close proximity to the LH1 α2-polypeptides in the transmembrane region (Fig. S6). Most of the interacting residues in the α2-polypeptide are unique among the LH1 α-polypeptides, and thus they likely play an important role in assembly of the LH1–RC complex.

### Ca^2+^-binding motif of the *A. tepidum* LH1 α1-polypeptide

**Figure 3:**
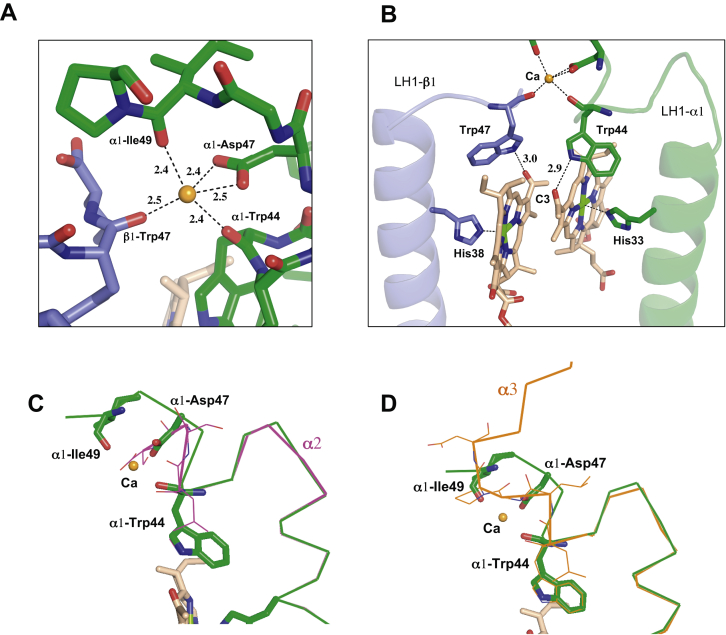
**Ca**^**2+**^**- and BChl *a*-binding sites in the *Allochromatium tepidum* LH1 complex.***A*, a typical Ca^2+^-binding site in LH1. Color scheme: LH1-α, *green*; LH1-β, *light-blue*; Ca^2+^, *orange ball*. *B*, a typical BChl *a*-binding site in an α1/β1 back-to-back subunit showing hydrogen bondings around the BChl *a* C3-acetyl groups. *C*, superposition of Cα carbons of the α1-polypeptide with that of α2-polypeptides showing that the C-terminus of the α2-polypeptide disrupts the Ca^2+^-binding site. *D*, superposition of Cα carbons of the α1-polypeptide with that of α3-polypeptide showing that the C-terminal region of the α3-polypeptide adopts a different conformation from that of the α1-polypeptide around the Ca-binding site. All distances are in ångstrom. BChl, bacteriochlorophyll; LH1, light-harvesting complex.

Due to relatively low sequence similarities (Fig. S5*C*), we were able to distinguish all three forms of the α-polypeptides in the *A. tepidum* LH1 from the cryo-EM density map: six α1, nine α2, and one α3 (Fig. 2, *A* and *B*). Only α1-polypeptides bind Ca ions using their mainchain oxygen atoms of Trp44 and Ile49 and sidechain carboxyl group of Asp47 as ligands (Figs. 2*C* and 3*A*). This WxxDxI Ca^2+^-binding motif is not present in the *A. tepidum* LH1 α2- and α3-polypeptides (Fig. 2*D*). However, comparative analyses showed that the motif is conserved in all Ca^2+^-bound LH1 α-polypeptides of *T. tepidum* (8) and *Trv.* strain 970 (14) (Fig. 2*E*). The Ile in this motif is essential because the corresponding residue in the *A. vinosum* LH1 α1-polypeptide is a Val (Fig. 2*E*), and the LH1 does not bind Ca^2+^ (18). These data indicate that the amino acid sidechain is important for Ca^2+^-binding even though the sidechain is not a direct ligand.

The Ca^2+^-binding sites formed by *A. tepidum* LH1 α1/β1- and α1/β3-pairs are located close to the LH1 BChl *a* molecules. The Ca^2+^-coordinating residues of α1-Trp44 and β-Trp47 also form hydrogen bonds with the C3-acetyl group of BChl *a* (Fig. 3*B*). Superpositions of the α1-polypeptide with α2- and α3-polypeptides revealed that the C-terminus of the shortest α2-polypeptide would disrupt the Ca-binding site (Fig. 3*C*) while the longest α3-polypeptide adopted a different conformation from that of the α1-polypeptide around the Ca-binding site (Fig. 3*D*). As a result, both α2- and α3-polypeptides are unable to bind Ca^2+^. Despite this specificity of Ca^2+^-binding, its effect on overall LH1 BChl *a* organization is likely limited. Only torsion angles of the C3-acetyl group are affected and tend to have smaller values for the BChl *a* associated with Ca^2+^-binding polypeptides than those that do not bind Ca^2+^ (Fig. S7). The torsion angle is proposed to correlate with the Q_y_ excitation energy (19, 20). There are no apparent differences between the Ca^2+^-bound and free LH1-αβ pairs in terms of the Mg–Mg distances between BChls *a*, the length of the His–Mg(BChl *a*) coordination, and the length of the hydrogen bond for the Trp and BChl *a* C3-acetyl group. Comparisons of the Mg–Mg and His–Mg distances with those from other bacteria are given in Table S2.

### Arrangement of the multiple LH1 polypeptides

Amino acid sequences of the LH1 polypeptides encoded in the *A. tepidum* genome are highly similar to those of the corresponding *A. vinosum* LH1 polypeptides (Fig. S8) (17). Among them, α2-, β1-, and β2-polypeptides are identical, but the *A. tepidum* β2-polypeptide was not detected in the purified LH1 complex (Fig. S5). It is notable that the β2-polypeptide was also undetected in the *A. vinosum* LH1 (21). The α1-polypeptides of *A. tepidum* form face-to-face dimeric subunits specifically with β3-polypeptides whereas α2-polypeptides specifically pair with β1-polypeptides (Fig. 2*B*), indicating a mechanism of molecular recognition between the polypeptide chains. The α1/β3 pairs are mainly stabilized through π-π stacking and cation-π interactions in the N-terminal domains, while α2/β1 and α3/β1 pairs lack the cation-π interaction in the N-terminal domains (Fig. S9). The single α3-polypeptide is located at a position corresponding to the opening of the monomeric LH1 ring of the purple bacterium *Rhodobacter sphaeroides* (Fig. S10) (22) and forms a subunit with the β1-polypeptide, likely due to its higher sequence similarity with the α2-polypeptide (Fig. S8*B*). These α1/β3 and α2/β1 (including α3/β1) subunits roughly divided into two groups, respectively, and align on the two sides of the LH1 ellipse (Fig. 2*B*). The arrangement is characterized by specific interactions between the shortest LH1 α2-polypeptides and the RC L- and M-subunits in the transmembrane region (Fig. S6*B*).

**Figure 4:**
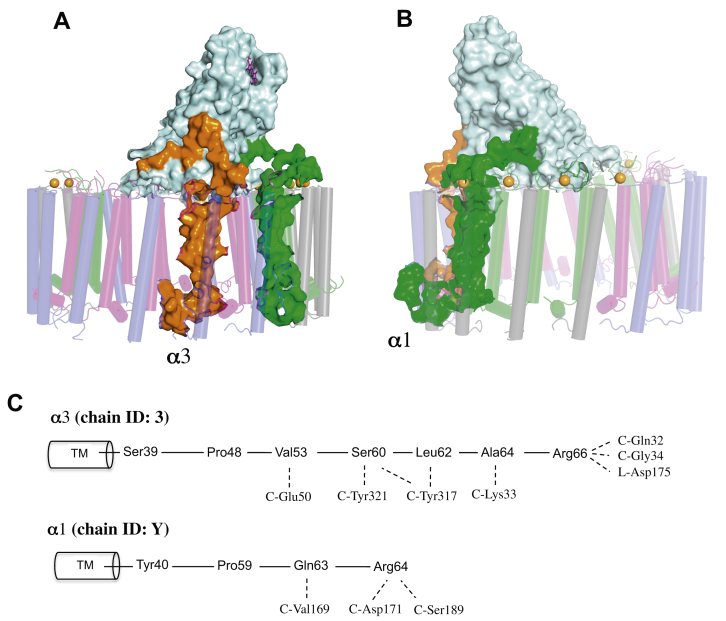
**Interactions between the *Allochromatium tepidum* LH1 α-polypeptides and the RC C-subunit.***A*, side view of the α3-polypeptide (*orange surface*) entangled with the RC C-subunit (*light-cyan surface*) through its long C-terminal domain. *B*, side view of the an α1-polypeptide (*green surface*) interacting with the RC C-subunit through its C-terminal domain. *C*, illustrations of interactions in the C-terminal domains of α3- and α1-polypeptides with the RC subunits. LH1, light-harvesting complex; RC, reaction center.

Although *A. tepidum* LH1 α1- and α3-polypeptides have no significant interactions with the RC subunits in the transmembrane region, these polypeptides have longer chain lengths and are able to interact with the RC C-subunit through their membrane-extruded C-terminal regions (Fig. 4*A*). The C-terminal domain of the α3-polypeptide is deeply entangled with the membrane-extruded N- and C-terminal regions of the C-subunit (Fig. 4). One of the α1-polypeptides (chain ID: Y) also interacts extensively with the RC C-subunit near the periplasmic surface (Fig. 4*B*). The membrane-extruded entity of the RC C-subunit is largely tilted toward one side of the LH1 ring where the α3-polypeptide and two of the α1-polypeptides (chain ID: Y and W in Fig. 2*B*) are located. Together with the characteristic position of the LH1 α2-polypeptides, the results lead to the conclusion that the unique arrangement of the *A. tepidum* LH1 α-polypeptides is defined by specific interactions with the RC subunits.

### An insertion/deletion in the LH1 α-polypeptides

**Figure 5:**
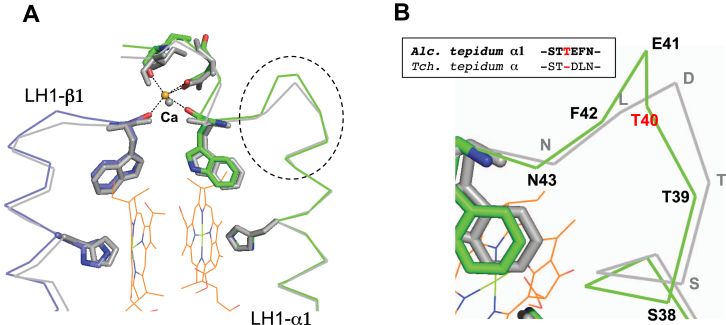
**An insertion in the *Allochromatium tepidum* α1-polypeptides.***A*, superposition of Cα carbons of the *Allochromatium tepidum* α1- and β1-polypeptides (*color*) with the corresponding *Thermochromatium tepidum* α- and β-polypeptides (*gray*) around the Ca^2+^- and BChl *a*-binding sites. Key residues that ligate to Ca^2+^ and BChl *a* are shown in *sticks*. *B*, expanded view of the loop region marked by the *dashed line* in (*A*) shows the position of the Thr40 insertion (*red*) and the alignment of sequences between *A. tepidum* and *T. tepidum*. BChl, bacteriochlorophyll.

Alignment of amino acid sequences between the *A. tepidum* Ca^2+^-bound α1-polypeptide and *T. tepidum* α-polypeptide revealed an insertion (Thr40) in the C-terminal region of the *A. tepidum* α1-polypeptide (Fig. 2*E*). A similar insertion (Ala) is also present at the same position in the *A. vinosum* LH1 α1-polypeptide. Because this position is close to both the BChl *a*-coordinating site within the membrane and the Ca^2+^-binding site on the membrane surface, the insertion was thought to influence the LH1 absorption maximum (Q_y_ transition) (2, 13, 23). Here, our structure of the *A. tepidum* LH1 shows that the insertion in the α1-polypeptide is located in a loop region immediately following the transmembrane α-helix (Fig. 5*A*). The effect of the inserted residue was minimized by forming a slightly more extended loop whose influence was essentially limited to a very narrow range (Thr39–Phe42) without significantly affecting both BChl *a*-coordinating and Ca^2+^-binding sites (Fig. 5*B*). Therefore, an insertion (in *A. tepidum* and *A. vinosum*) or deletion (in *T. tepidum*) in this loop region should not have a significant effect on the LH1-Q_y_ transition in any of these phototrophs. However, the small but distinct difference in the loop size between *A. tepidum* and *T. tepidum* suggests that the LH1 complex of the thermophilic *T. tepidum* may have evolved to adopt a shorter loop in this region in order to reduce thermal fluctuations and improve stability; such would help the *T. tepidum* LH1 to remain functional at temperatures above that supporting growth of *A. tepidum*.

## Discussion

The multiple α/β-containing LH1 complex of *A. tepidum* offers a unique model system to investigate the minimum structural requirement for Ca^2+^-binding in bacterial photosynthetic LH1 complexes. This was challenging with the fully Ca^2+^-bound LH1s reported previously from *T. tepidum* (8) and *Trv.* strain 970 (14) because all LH1 polypeptides in the complexes of these bacteria participated in Ca^2+^-binding and have similar amino acid sequences around the Ca^2+^-binding sites, making it difficult to distinguish the roles of individual residues. However, unlike the LH1 complexes from *T. tepidum* and *Trv.* strain 970, the *A. tepidum* LH1 complex contains both Ca^2+^-bound and Ca^2+^-free αβ-subunits in a single ring, allowing for an unambiguous identification of the key residues responsible for Ca^2+^-binding. Of the total 16 *A. tepidum* LH1 α-polypeptides, only six copies of the α1-polypeptide bind Ca^2+^ to form Ca^2+^-binding sites with β1- or β3-polypeptides. Three amino acids (Trp44, Asp47, and Ile49) in the C-terminal region of *A. tepidum* α1-polypeptide were identified as the Ca^2+^-ligating residues that form a Ca^2+^-binding WxxDxI motif. This structural motif represents the minimum requirement for an LH1 polypeptide to be able to bind Ca^2+^ and is conserved in all Ca^2+^-bound α-polypeptides with reported structures. It is notable that the WxxDxI motif is also present in the LH1 α-polypeptides of *Trv. winogradskyi* DSM 6702^T^ and *Lamprocystis purpurea* (*Amoebobacter purpureus*) DSM 4197^T^ (24). Whether the LH1 complexes from these purple sulfur bacteria bind Ca^2+^ is unknown, but the presence of this key motif is suggestive.

The partially Ca^2+^-bound structure of the *A. tepidum* LH1 well explains its intermediate properties in terms of thermostability, spectroscopy, and phototrophic growth (18) compared with the fully Ca^2+^-bound LH1 of the thermophilic *T. tepidum* and the Ca^2+^-free LH1 of the mesophilic *A. vinosum*. The native *A. tepidum* LH1–RC is much more thermostable than that of *A. vinosum* (18). The purified *A. tepidum* LH1–RC has an LH1-Q_y_ transition at 890 nm (Fig. S1) close to that of *A. vinosum* (884 nm) but far from that of *T. tepidum* (915 nm). The Ca^2+^-requirements for an LH1-Q_y_ redshift, LH1–RC thermostability, and phototrophic growth of *A. tepidum* were also less strict than those of *T. tepidum* (18). One of the important interactions underling these intermediate properties is the hydrogen bonding between BChl and LH1 polypeptides that is strongly affected by Ca^2+^-binding. The hydrogen bonding strengths between the BChl C3-acetyl group and the Trp residues in the C-termini of LH1 polypeptides measured by our Raman spectroscopy revealed good correlation with the LH1-Q_y_ position (18). That is, the fully Ca^2+^-bound *T. tepidum* LH1 forms the strongest hydrogen bonds and exhibits the largest Q_y_ redshift followed by the partially Ca^2+^-bound *A. tepidum* LH1; hydrogen bonding strength in the latter is much reduced from that of *T. tepidum* but is larger than that of the Ca^2+^-free *A. vinosum* LH1. These results can be well explained by our structure. The average hydrogen-bonding lengths between the BChl C3-acetyl oxygen and the Trp nitrogen (ε1) are 2.9 Å and 3.1 Å for the *A. tepidum* α- and β-polypeptides, respectively. These lengths are longer than the 2.8 Å and 2.9 Å measured for the corresponding *T. tepidum* α- and β-polypeptides (PDB: 5Y5S), confirming that the hydrogen bonds formed in *A. tepidum* LH1 are weaker than those in *T. tepidum*. The weakened hydrogen bonding incorporated with a partial Ca^2+^-binding in *A. tepidum* LH1 likely contribute to its relatively lower thermostability than that of *T. tepidum* LH1. Our structural information combined with biochemical and spectroscopic analyses indicates that these properties are correlated with the Ca^2+^ content of the LH1 and is consistent with previous conclusions that Ca ions tightly lock the LH1 rings by forming a Ca^2+^-connected network that strongly contributes to both thermostability and the LH1-Q_y_ redshift (8, 14). Our results also support the hypothesis that *A. tepidum* is a “transitional organism” in the sense of bridging the phenotypic gap between mesophilic and thermophilic purple sulfur bacteria. Besides evidence presented from comparative structural analyses of LH1–RC complexes, this conclusion is supported by comparative physiological and genomic studies of *A. tepidum* (17).

Effects of charge distribution near the BChl molecules on the LH1-Q_y_ have been well investigated (19, 25, 26, 27). There is a correlation between the direction of the Q_y_ change and the location and sign of the point charge: positive charges near pyrrole ring I of BChl or negative charges near ring III result in large red-shifts, whereas reversed charges at these locations cause blue-shifts (26, 28, 29, 30). The Ca ions in *A. tepidum* and *T. tepidum* LH1 are located at positions with a distance of ca. 11 Å from the rings I of BChl *a* (Figs. 3*B* and 5*A*), as measured from the BChl C3-acetyl oxygen. Substitution experiments revealed that these Ca ions indeed alter the electrostatic interaction network and in doing so, influence the LH1 Q_y_ transition (30). On the other hand, energy transfer from the low-energy LH1 BChl *a* to the RC has also been investigated mainly using the largely redshifted *T. tepidum* LH1 and its derivatives (31, 32, 33). Despite an energetically “uphill” process, the energy transfer rate has been shown to be comparable to those for other phototrophic bacteria that show “normal” LH1-Q_y_ absorptions at ∼880 nm, indicating that efficiency of energy transfer from the redshifted LH1 to RC is not significantly compromised by these low-energy pigments.

The arrangement of multiple α/β-polypeptides within the *A. tepidum* LH1 ring is also unique. It differs from that of another multiple α/β-containing LH1 complex from *Trv.* strain 970 (14) in (i) higher specificity of pairing of the α/β-subunit and (ii) grouping of the α-polypeptides around the RC. The *A. tepidum* α1-polypeptides specifically pair with β3-polypeptides to form face-to-face subunits, and similarly the α2-polypeptides specifically pair with β1-polypeptides (Figs. 2*B* and S10*A*). There are no α1/β1- and α2/β3-subunits in the *A. tepidum* LH1 complex. By contrast, multiple copies of both α1- and α3-polypeptides in *Trv.* strain 970 form subunits with either β1- or β4-polypeptides (Fig. S10*B*) (14). The multiple α1/β3 and α2/β1 pairs in *A. tepidum* LH1 form two groups, respectively, and are positioned on the opposite sides of the RC core with the α2-polypeptides in close proximity to the RC L- and M-subunits in the transmembrane region. By contrast, all α1-polypeptides in *Trv.* strain 970 are aligned on one side of the RC and all α3-polypeptides are located on the opposite side with the single copy of α2- and α4-polypeptides in close proximity to the RC in the transmembrane region (Fig. S10*B*). In the *A. tepidum* LH1, a split of the clustered Ca^2+^-bound α1/β3(β1) pairs into two groups may also weaken the effects of Ca^2+^-binding on thermostability and LH1-Q_y_ because both properties are highly collective over all αβ pairs in the LH1 ring. The characteristic arrangement of the *A. tepidum* LH1 αβ-polypeptides is likely regulated in its expression and/or assembly processes through multiple specific interactions within the LH1 complex (among α- and β-polypeptides through N-terminal domains and/or Ca^2+^-binding) and between LH1 and the RC (α-polypeptides with the RC L- and M-subunits in the transmembrane regions and C-terminal domain of the α-polypeptide with the RC C-subunit).

In our cryo-EM structure of the *A. tepidum* LH1 complex, an amino acid insertion of (Thr40) in the C-terminal region of the Ca^2+^-bound α1-polypeptide turned out to have no significant effect on structures near the BChl *a*-coordinating and Ca^2+^-binding sites (Fig. 5). On the one hand, this implies that the deletion of this residue in *T. tepidum* α-polypeptides may not be a decisive factor for its LH1-Q_y_ redshift at 915 nm but rather a mechanism to enhance thermostability. On the other hand, insertion of an Ala at the corresponding position in the *T. tepidum* α-polypeptides resulted in a mutant strain whose LH1 exhibited an absorption maximum at 899 nm (23). These somewhat conflicting results could be explained by either of two or both possibilities: (i) the genetically modified *T. tepidum* α-polypeptides may adopt a different conformation in the loop region from that of the native *A. tepidum* LH1 α1-polypeptide, causing structural changes at the BChl *a*-coordinating and/or Ca^2+^-binding sites, and (ii) the inserted Ala in the mutant *T. tepidum* LH1 α-polypeptide may have a different effect from that of Thr in the *A. tepidum* LH1 α1-polypeptide as seen in the Ca^2+^-binding motif where the sidechain was shown to play an important role for Ca^2+^-binding (Fig. 2*E*). To clarify this issue, structural details would be required on the native *A. vinosum* LH1 complex (Fig. 2*E*).

## Experimental procedures

### Preparation and characterization of the LH1–RC complex

Cells of *A. tepidum* were cultivated phototrophically (anoxic/light) at 43 °C for 7 days under incandescent light (60W). Preparation of the *A. tepidum* LH1–RC followed the procedures described previously (18) with minor modifications. The solubilized crude LH1–RC solution was loaded on a DEAE column (Toyopearl 650S, TOSOH) equilibrated with 20 mM Tris–HCl (pH 7.5) and 0.1% *n*-dodecyl β-D-maltopyranoside at 7 °C. LH1–RC components were eluted by a linear gradient of CaCl_2_ from 10 mM to 50 mM, and fractions with A_890_/A_280_ > 2.2 were collected for the subsequent measurements (Fig. S1), then assessed by negative-stain EM using a JEM-1011 instrument (JEOL). Masses and composition of the LH1 polypeptides were measured by MALDI-TOF/MS and reversed-phase HPLC, respectively, using methods described elsewhere (34). Phospholipid and quinone contents of purified LH1–RC were analyzed (Fig. S11) as previously described (35, 36).

### Cryo-EM data collection

Proteins for cryo-EM were concentrated to ∼3 mg/ml. Three microliters of the protein solution were applied on a glow-discharged holey carbon grids (200 mesh Quantifoil R2/2 molybdenum), which had been treated in a DII-29020HD (JEOL) for 40 s, and then plunged into liquid ethane at –178 °C using an EM GP2 plunger (Leica, Microsystems). The applied parameters were blotting time 6s and 90% humidity at 4 °C. The data were collected on a CRYO-ARM300 (JEOL) electron microscope at 300 kV equipped with a K3 camera (Gatan). An in-column energy filter with a slit width of 20 eV was inserted for acquisition of movie frames. The movies were recorded using JADAS software (JEOL) (37) at a nominal magnification of 60K in counting mode and a pixel size of 0.814 Å at the specimen level with a dose rate of 17.7 e-per physical pixel per second, corresponding to 26.7 e-per Å^2^ per second at the specimen level. The exposure time was 1.5 s, resulting in an accumulated dose of 40.0 e-per Å^2^. Each movie includes 20 fractioned frames.

### Image processing

All of the stacked frames were subjected to motion correction with MotionCor2 (38), and defocus was estimated using CTFFIND4 (39). A total of 309,706 particles were selected from 1901 micrographs using the EMAN2 suite (40). The initial 3-D model was generated with 63,844 particles from 685 selected micrographs with underfocus values ranging between 2 and 3 μm using RELION3.0 (41). The particles were further analyzed with RELION3.0 (41), and 227,955 particles were selected by 2-D classification and divided into four classes by 3-D classification resulting in only one good class containing 158,474 particles. Then, 156,992 particles were further chosen based by 2-D classification. The 3-D auto refinement without any imposed symmetry (C1) produced a map at 2.93 Å resolution after contrast transfer function refinement, Bayesian polishing, masking, and postprocessing. The selected 156,992 particle projections were subjected to subtraction of the detergent micelle density followed by 3-D auto refinement to yield the final map with a resolution of 2.81 Å according to the gold-standard Fourier shell correlation using a criterion of 0.143 (Fig. S2) (42). The local resolution maps were calculated on RESMAP (43).

### Model building and refinement of the LH1–RC complex

The atomic model of the *T. tepidum* LH1–RC (PDB code 5Y5S) was fitted to the cryo-EM map obtained for the *A. tepidum* LH1–RC using Chimera (44). Amino acid substitutions and real space refinement for the peptides and cofactors were performed using COOT (45). The C-terminal regions of the LH1 α3-subunit were modeled *ab-initio* based on their density. The manually modified model was real-space-refined on PHENIX (46), and the COOT/PHENIX refinement was iterated until the refinements converged. Finally, the statistics calculated using MolProbity (47) were checked. Figures were drawn with the Pymol Molecular Graphic System (Schrödinger) (48) and UCSF Chimera (44).

## Data availability

Map and model have been deposited in the EMDB and PDB with the accession codes: EMD-32100 and PDB-7VRJ. All other data are available from the authors upon reasonable request.

## Supporting information

This article contains supporting information.

## Conflict of interest

Authors K. K., N. H., I. I., and Y. O. are employees of JEOL Ltd. This does not alter the authors’ adherence to all policies on sharing data and materials. The authors declare that they have no conflicts of interest with the contents of the article.
