# Supplementary material for: A Ca2+-binding motif underlies the unusual properties of certain photosynthetic bacterial core light-harvesting complexes
Source: J Biol Chem. 2022 Apr 20;298(6):101967. doi: 10.1016/j.jbc.2022.101967 (PMC9133646; doi:10.1016/j.jbc.2022.101967)
Supplement: Supplemental Tables S1–S2 and Figures S1–S11 [file mmc1.pdf]

Supporting Information

A Ca^{2+}-binding motif underlies the unusual properties of certain photosynthetic bacterial core light-harvesting complexes

K. Tani[1,‖,*], K. Kobayashi[2,‖], N. Hosogi[2], X.-C. Ji[3], S. Nagashima[4], K. V. P. Nagashima[4], A. Izumida[5], K. Inoue[4,5], Y. Tsukatani[6], R. Kanno[7], M. Hall[7], L.-J. Yu[8], I. Ishikawa[2], Y. Okura[2], M. T. Madigan[9], A. Mizoguchi[1], B. M. Humbel[7], Y. Kimura[10,*] & Z.-Y. Wang-Otomo[3,*]

Corresponding authors: K. Tani, Y. Kimura and Z.-Y. Wang-Otomo

E-mail: ktani@doc.medic.mie-u.ac.jp; ykimura@people.kobe-u.ac.jp; wang@ml.ibaraki.ac.jp

This file contains:

(1) Tables S1 to S2

(2) Figures S1 to S11

Table S1. Cryo-EM data collection, refinement and validation statistics of the *Alc. tepidum* LH1-RC complex

	LH1-RC complex (EMDB-32100) (PDB-7VRJ)
**Data collection and processing**	
Magnification	60000
Voltage (kV)	300
Electron exposure (e–/Å^2)	40
Defocus range (μm)	–1.0 to –3.0
Calibrated pixel size (Å)	0.814
Detector physical pixel size (μm)	5
Symmetry imposed	C1
Initial particle images (no.)	309706
Final particle images (no.)	156992
Map resolution (Å)	2.8
FSC threshold	0.143
Map resolution range (Å)	326–2.8
**Refinement**	
Initial model used (PDB code)	5Y5S
Model resolution (Å)	2.9
FSC threshold	0.5
Model resolution range (Å)	140–2.8
Map sharpening *B* factor (Å^2)	–62
Model composition	
Non-hydrogen atoms	26309
Protein residues	604
Ligands	114
B factors (Å^2)	
Protein	39.9
Ligand	38.2
R.m.s. deviations	
Bond lengths (Å)	0.006
Bond angles (°)	2.767
Validation	
MolProbity score	1.77
Clashscore	9.32
Poor rotamers (%)	1.43
Ramachandran plot	
Favored (%)	97.10
Allowed (%)	2.86
Disallowed (%)	0.04

Table S2. Comparison of the distances of His–BChl(Mg) and BChl(Mg)–BChl(Mg) in LH1, LH2 and RC special pairs from various phototrophic bacteria.

	Distance of His(Nε2) to BChl–Mg (Å)[a]		Distance of Mg–Mg (Å)[a]	
LH1 or LH2	α	β	Long	Short
Alc. tepidum **(LH1)**	**2.54**	**2.29**	**9.52**	**8.75**
Rba. sphaeroides (LH1)	2.58	2.20	9.55	8.37
Rsp. rubrum (LH1)	2.27	2.03	9.34	8.51
Rps. palustris (LH1-W)	2.93	2.71	9.61	8.29
Tch. tepidum (LH1)	2.19	2.19	8.88	8.72
Trv. strain 970 (LH1)	2.61	2.32	8.90	8.46
Blc. viridis (LH1)	2.54	2.25	8.8	8.5
Rfx. castenholzii (B880)	2.32	2.29	9.5	9.3
Rbl. acidophilus (B850)	2.34	2.34	9.5	8.8
Phs. molischianum (B850)	2.27	2.32	9.2	8.9
RC (special pair)	L-subunit	M-subunit	BChl *a* (L)–BChl *a* (M)	
Alc. tepidum	**2.44**	**2.49**	**7.97**	
Rba. sphaeroides (LH1-RC)	2.21	2.11	7.79	
Rba. sphaeroides (RC-only)	2.27	2.06	7.84	
Rsp. rubrum	2.09	2.12	7.76	
Rps. palustris	2.73	2.74	7.69	
Tch. tepidum	2.17	2.19	7.87	
Trv. strain 970	2.33	2.31	7.65	
Blc. viridis	2.36	2.35	7.83	

[a] These values were derived from Protein Data Bank: 7EQD for *Rsp. rubrum*, 5Y5S for *Tch. tepidum*, 7C9R for *Trv.* strain 970, 6Z5S for *Rps. palustris*, 6ET5 for *Blc. viridis*, 5YQ7 for *Rfx. castenholzii*, 1NKZ for *Rbl. acidophilus* (previously *Rps. acidophila*), 1LGH for *Phs. molischianum*, 2J8C for *Rba. sphaeroides* (RC-only).

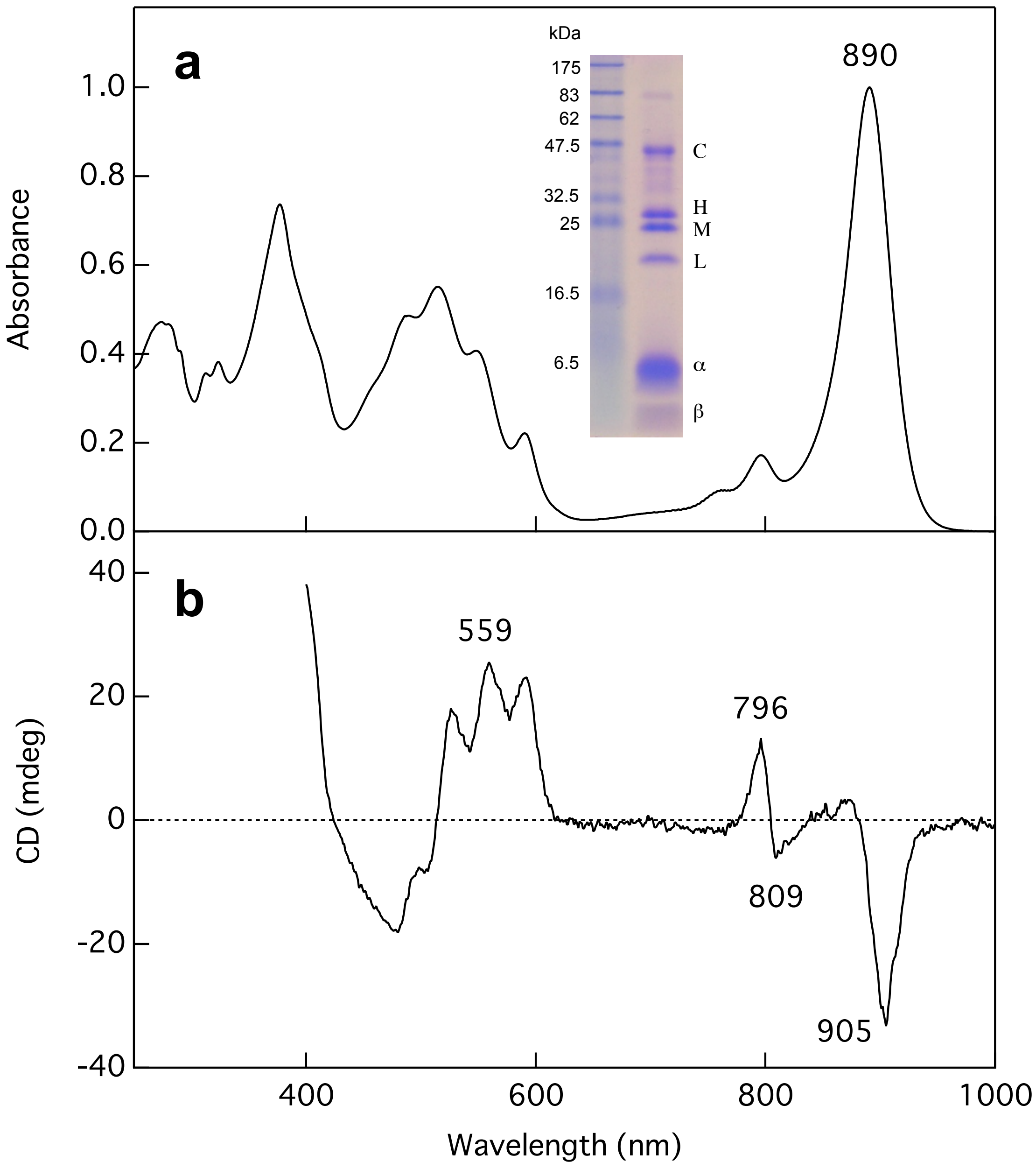

Figure S1. Absorption (a) and circular dichroism (b) spectra of the purified *Alc. tepidum* LH1-RC complex at room temperature. Inset: Coomassie blue-stained 12% SDS-PAGE gel for the purified LH1-RC with their assignments indicated. CD spectrum was recorded on a Jasco J-720w spectropolarimeter in the range from 400 nm to 1000 nm under the following conditions: 100 nm/min scan speed, 5 nm bandwidth, 1 sec response time, 5 scans.

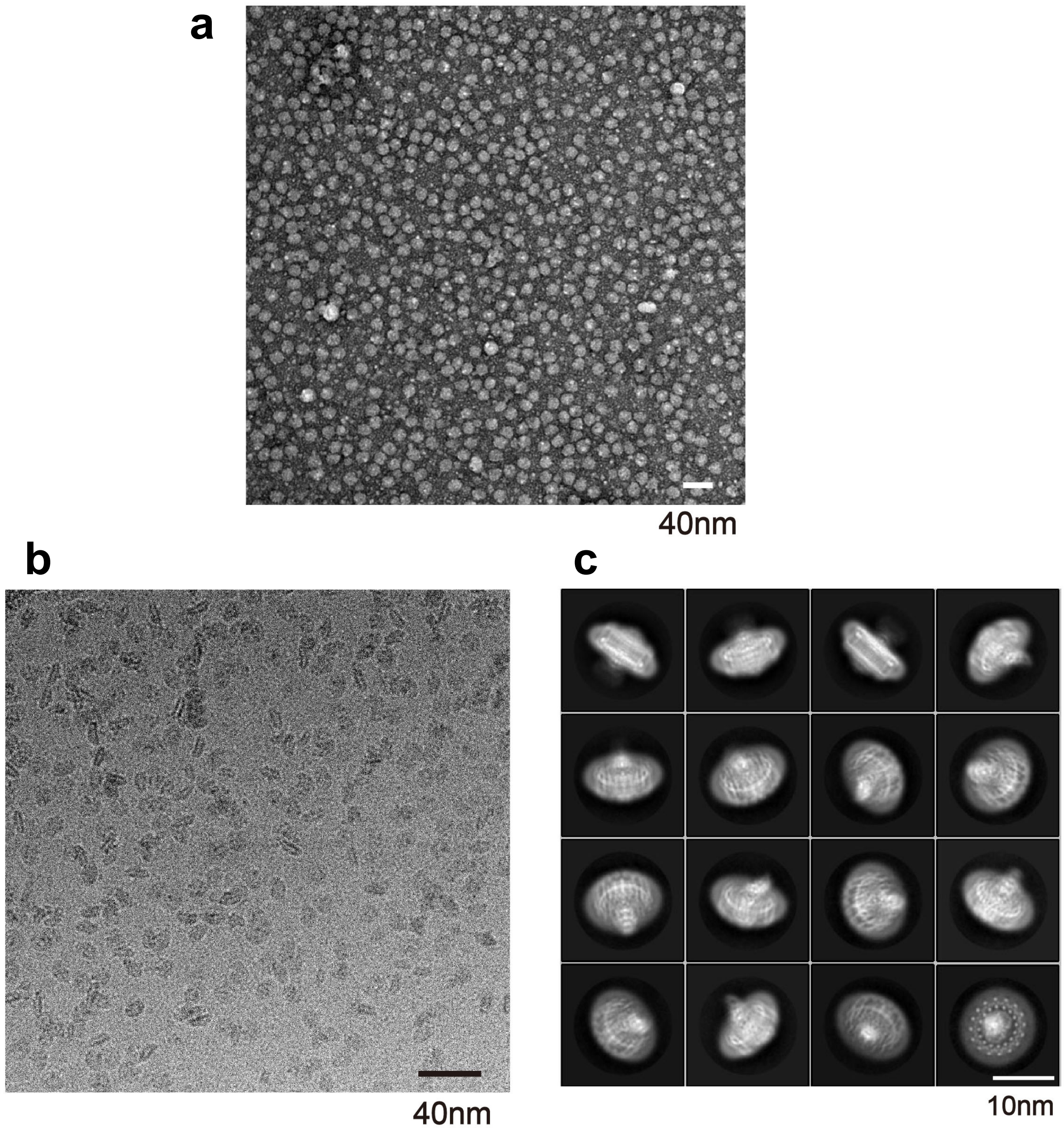

Figure S2. Electron micrographs of the *Alc. tepidum* LH1-RC complex. A representative negatively stained **(a)** and cryo-EM **(b)** micrographs of the purified LH1-RC particles. **(c)** Representative 2D class averages from cryo-EM micrographs.

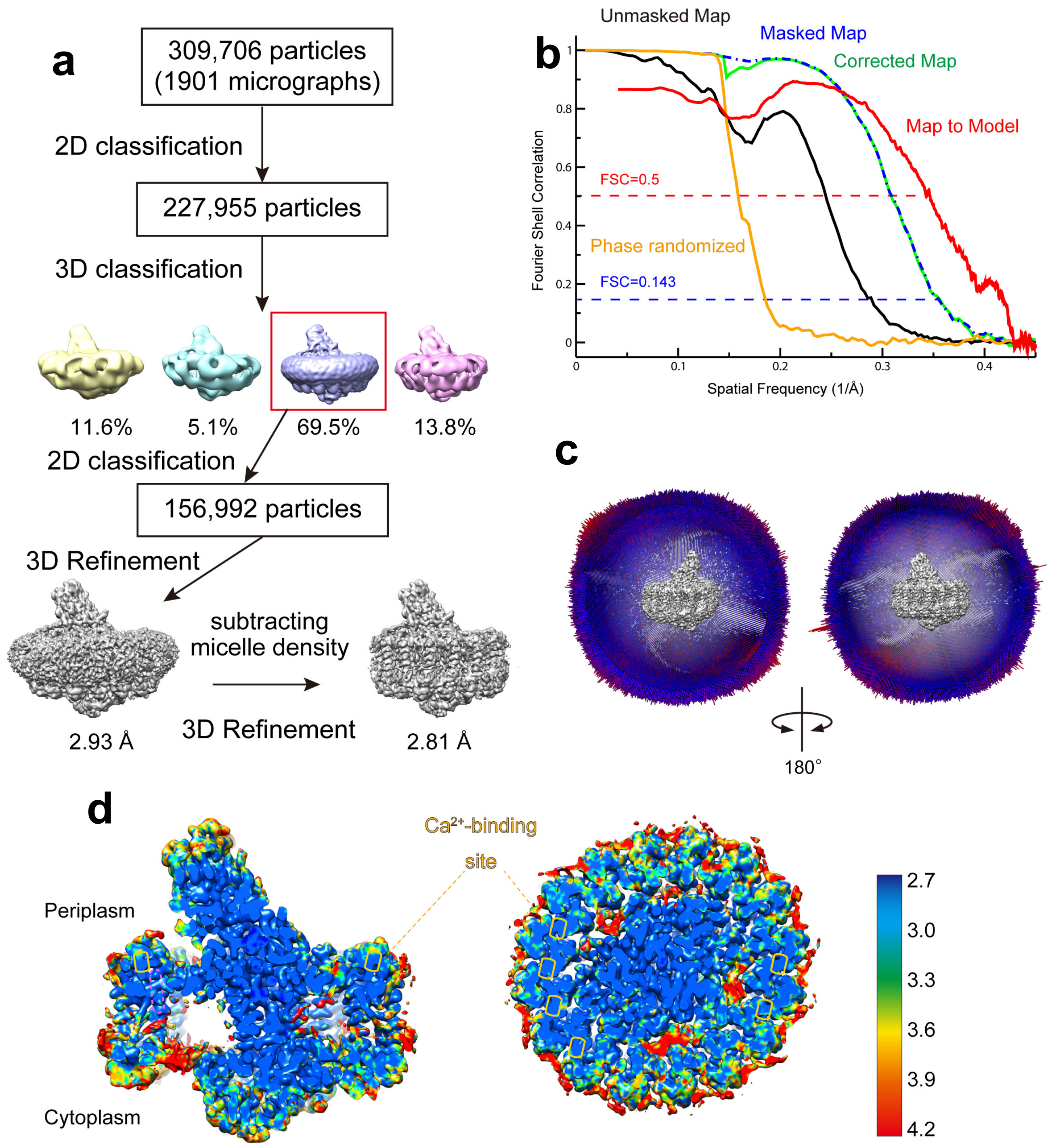

Figure S3. Structure determination of the *Alc. tepidum* LH1-RC complex by cryo-EM. **(a)** Image processing flow of 3D classification and reconstruction. **(b)** The Fourier shell correlation (FSC) plots of the cryo-EM map (unmasked: black, masked: blue, phase randomized corrected: green, phase randomized: orange) and the FSC plot of the model versus the final map (red) are superimposed. **(c)** Angular distribution of reconstructed particles. **(d)** Local resolution representation of the LH1-RC structure. A longitudinal sectional view (*left*) and a central cross sectional view (*right*) from periplasmic side. The map is shown in rainbow colors as shown in the right color bar. The contour level of the map raw density is 0.005.

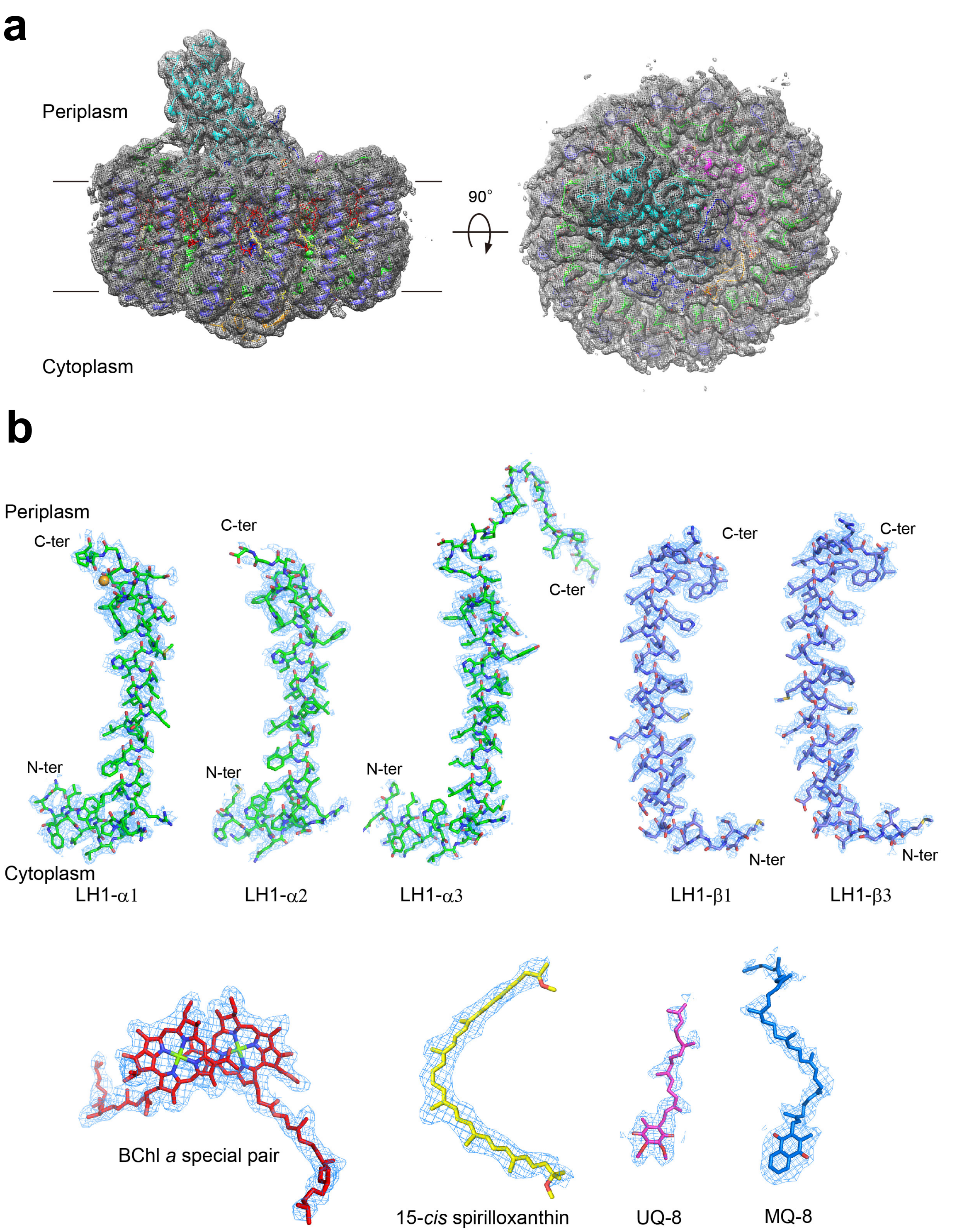

Figure S4. Cryo-EM densities and structural models in the *Alc. tepidum* LH1-RC complex. (a) Overall structure of the LH1-RC complex. Cartoon representation of the complex with the cryo-EM density in gray mesh. Side view (*left*) parallel to the membrane plane and top view (*right*) from periplasmic side. **(b)** Selected polypeptides and cofactors. The density maps are shown at a contour level of 4.0σ, except for the UQ-8 and MQ-8 (3.0σ). The color codes are the same as in Fig. 1.

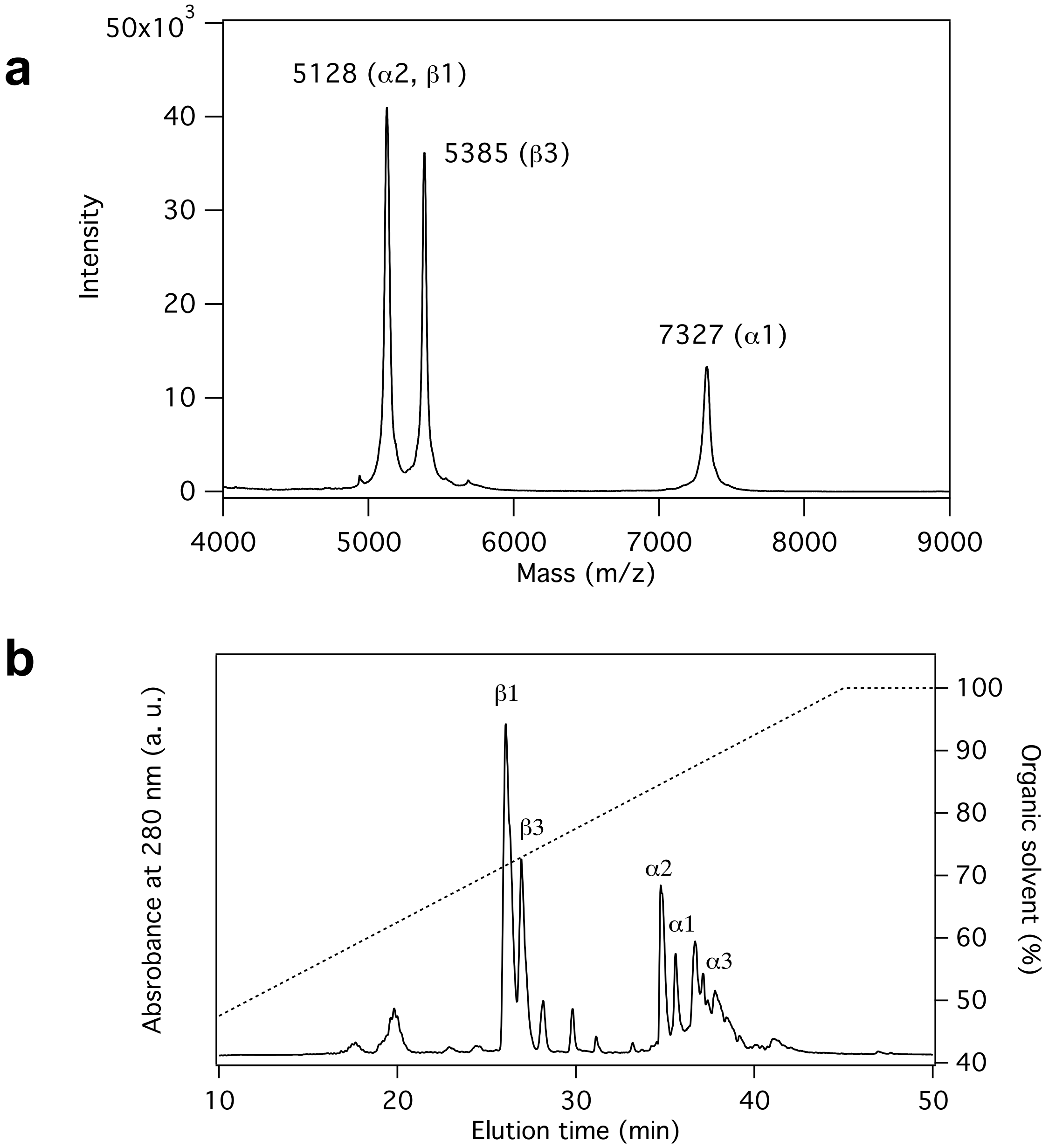

c

α1 formyl-MSPDLWKIWLLIDPRRVLIAVFAFLTILGLAIHMILLSTTEFNWLEDGIPAAKVQQVTPVVPQR (Mw=7325)
α2 formyl-MHKIWQIFDPRRTLVALFGFLFVLGLLIHFILLSSPAFNWLSGS (Mw=5131)
α3 MPQQLYKIWLAFDPRMALIGLGAFLFALALFIHYMLLSSPGFDWLLGPDHAPVALSAGMSALPAGR (Mw=7188)

β1 ANSSMTGLTEQEAQEFHGIFVQSMTAFFGIVVIAHILAWLWRPWL (Mw=5135)
β2 MANENRSMSGLTEDEAREFHGIFVSSFVVFTGIVVVAHILVWLWRPWL
β3 ADQKSMTGLTEDEAKEFHGIFTQSMTMFFGIVIIAHILAWLWRPWL (Mw=5368)

Figure S5. Characterization of the *Alc. tepidum* LH1 polypeptides. (a) MALDI-TOF/MS spectrum of the purified LH1-RC obtained under the same conditions as described in the Materials and Methods section. α2- and β1-polypeptides were not resolved. **(b)** Reverse-phase HPLC chromatograms (TSKgel, SuperODS, 4.6×100 mm, TOSOH) of the purified LH1-RC eluted at 25°C by a gradient of 40–100% organic solvent consisted of acetonitrile/2-propanol (2:1) containing 0.1% trifluoroacetic acid. **(c)** Amino acid sequences of the expressed LH1 αβ-polypeptides with calculated molecular weight (Mw). The β2-polypeptide (gray fonts) was not detected.

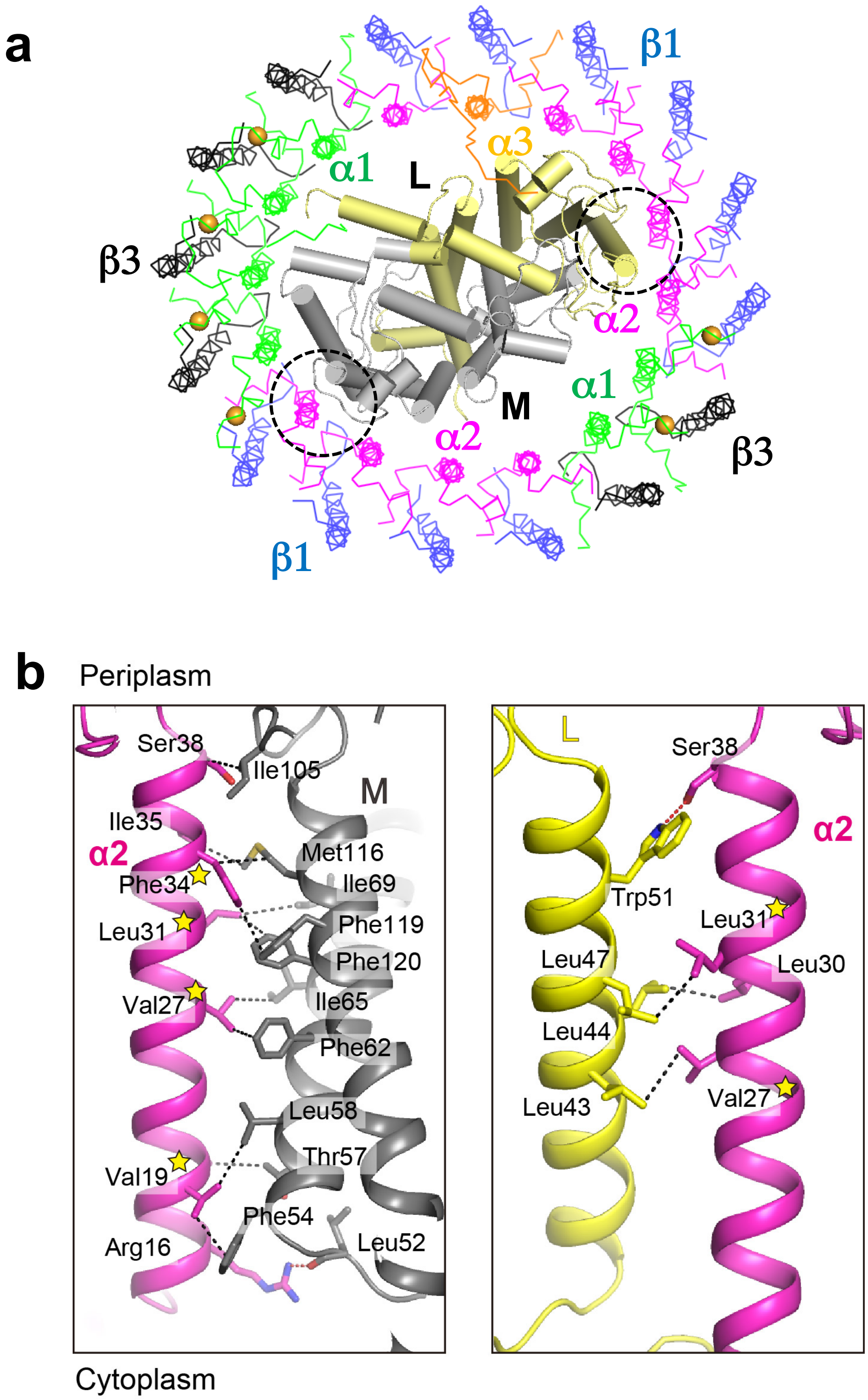

Figure S6. Interactions between LH1/RC of *Alc. tepidum*. (a) Interacting sites (dashed circles) between LH1 α2-polypeptides (magenta) and the RC L(light-yellow)- and M(gray)-subunits. Color scheme is same as in Fig. 2a. **(b)** Interactions in the transmembrane region between LH1 α2(magenta)- and M(gray) or L(yellow)-polypeptides. Dashed lines indicate distances in a range of 2.6–4.5 Å. The star symbol indicates an unique residue of α2 among α-polypeptides. Residue numbers for α2 are indicated as offset of +4 from the N-terminus Met.

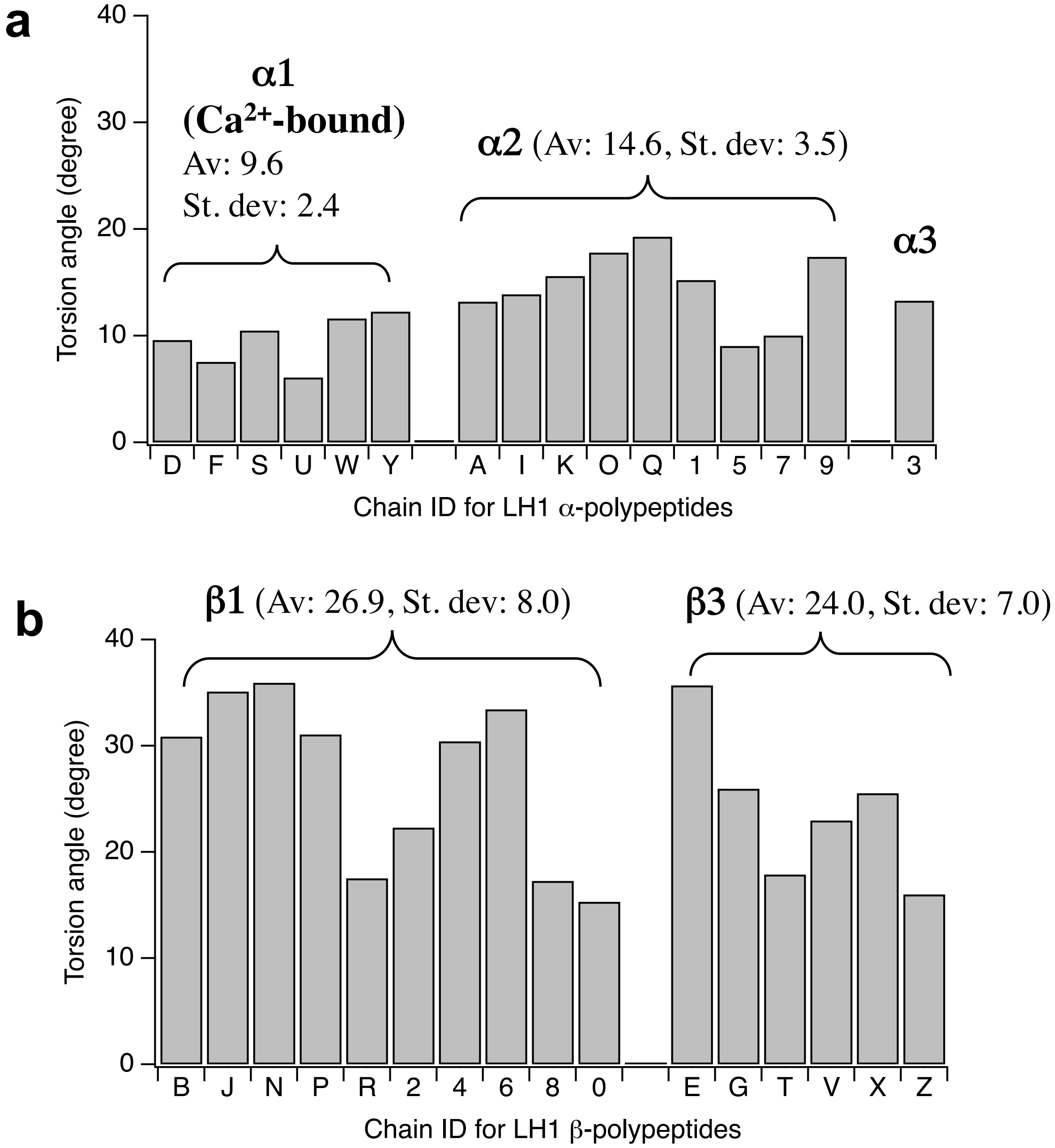

Figure S7. Torsion angle distribution of the BChl *a* C3-acetyl group in *Alc. tepidum* LH1. (a) LH1 α-polypeptides. **(b)** LH1 β-polypeptides.

a

pufA	
A.tepidum A1	MSPDLWKIWLLIDPRRVLIAVFAFLTILGLAIHMILLSTTEFNWLEDGIPAAKVQQVTPVVPQR
A.vinosum A1	MSPDLWKIWLLVDPRRILIAVFAFLTVLGLAIHMILLSTAEFNWLEDGVPAATVQQVTPVVPQR
A.tepidum A2	MHKIWQIFDPRRTLVALFGFLFVLGLLIHFILLSSPAFNWLSGS
A.vinosum A2	MHKIWQIFDPRRTLVALFGFLFVLGLLIHFILLSSPAFNWLSGS
A.tepidum A3	MPQQLYKIWLAFDPRMALIGLGAFLFALALFIHYMLLSSPGFDWLLGPDHAPVALSAGMSALPAGR
A.vinosum A3	MMPQLYKIWLAFDPRMALIGLGAFLFALALFIHYMLLRSPEFDWLLGPDYAPVTLSAGMSALPAGR
pufB	
A.tepidum B1	MANSSMTGLTEQEAQEFHGIFVQSMTAFFGIVVIAHILAWLWRPWL
A.vinosum B1	MANSSMTGLTEQEAQEFHGIFVQSMTAFFGIVVIAHILAWLWRPWL
A.tepidum B2	MANENRSMSGLTEDEAREFHGIFVSSFVVFTGIVVVAHILVWLWRPWL
A.vinosum B2	MANENRSMSGLTEDEAREFHGIFVSSFVVFTGIVVVAHILVWLWRPWL
A.tepidum B3	MADQKSMTGLTEDEAKEFHGIFTQSMTMFFGIVIIAHILAWLWRPWL
A.vinosum B3	MADQKSMTGLTEEEAKEFHGIFTQSMTMFFGIVIIAHILAWLWRPWL

b ***Alc. tepidum*** **α- and β-polypeptides**

	α2	α3
α1	**57**	39
α2		**57**

	β2	β3
β1	72	**80**
β2		66

Figure S8. Sequence comparison of the LH1 polypeptides between *Alc. tepidum* and *Alc. vinosum*, and of *Alc. tepidum*. **(a)** Alignments of amino acid sequences encoded in the *Alc. tepidum* and *Alc. vinosum* genomes for each PufA and PufB pairs. Differences of the residues between the two species are indicated by red fonts. **(b)** Comparison of the sequence similarities between the α- and β-polypeptides of *Alc. tepidum* using sequence alignment scores from the CLUSTAL 2.1.

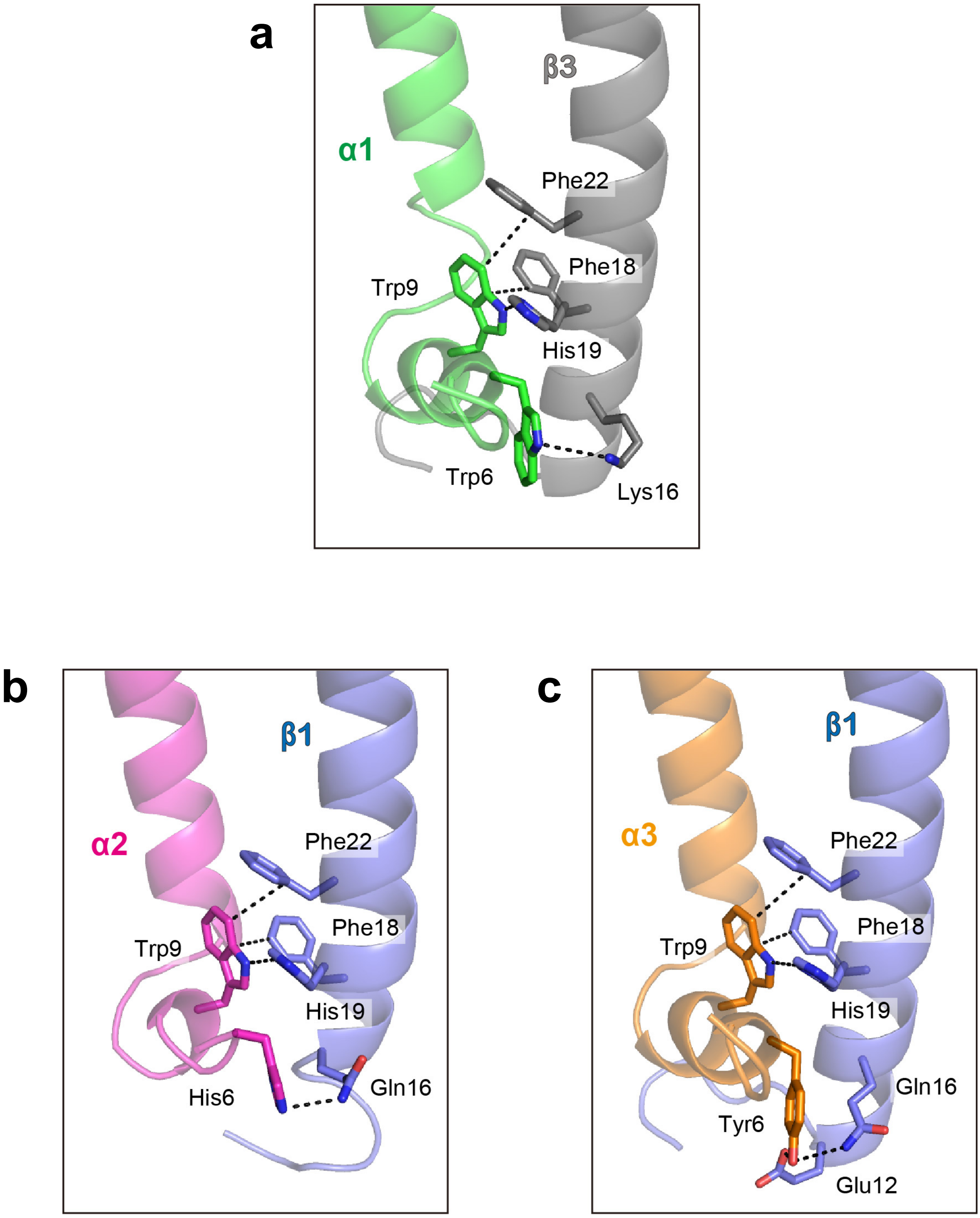

Figure S9. Interactions between LH1 α/β-polypeptides of *Alc. tepidum*. Interactions in the N-terminal domains between LH1 α1(green)- and β3(gray)-polypeptides **(a),** between LH1 α2(magenta)- and β1(slate bule)-polypeptides **(b)**, and between LH1 α3(orange)- and β1(slate bule)-polypeptides **(c)**. Dashed lines indicate distances in a range of 2.6–4.2 Å. Residue numbers for α2 and β1 are indicated as offsets of +4 and +1, respectively, from the N-terminus Met.

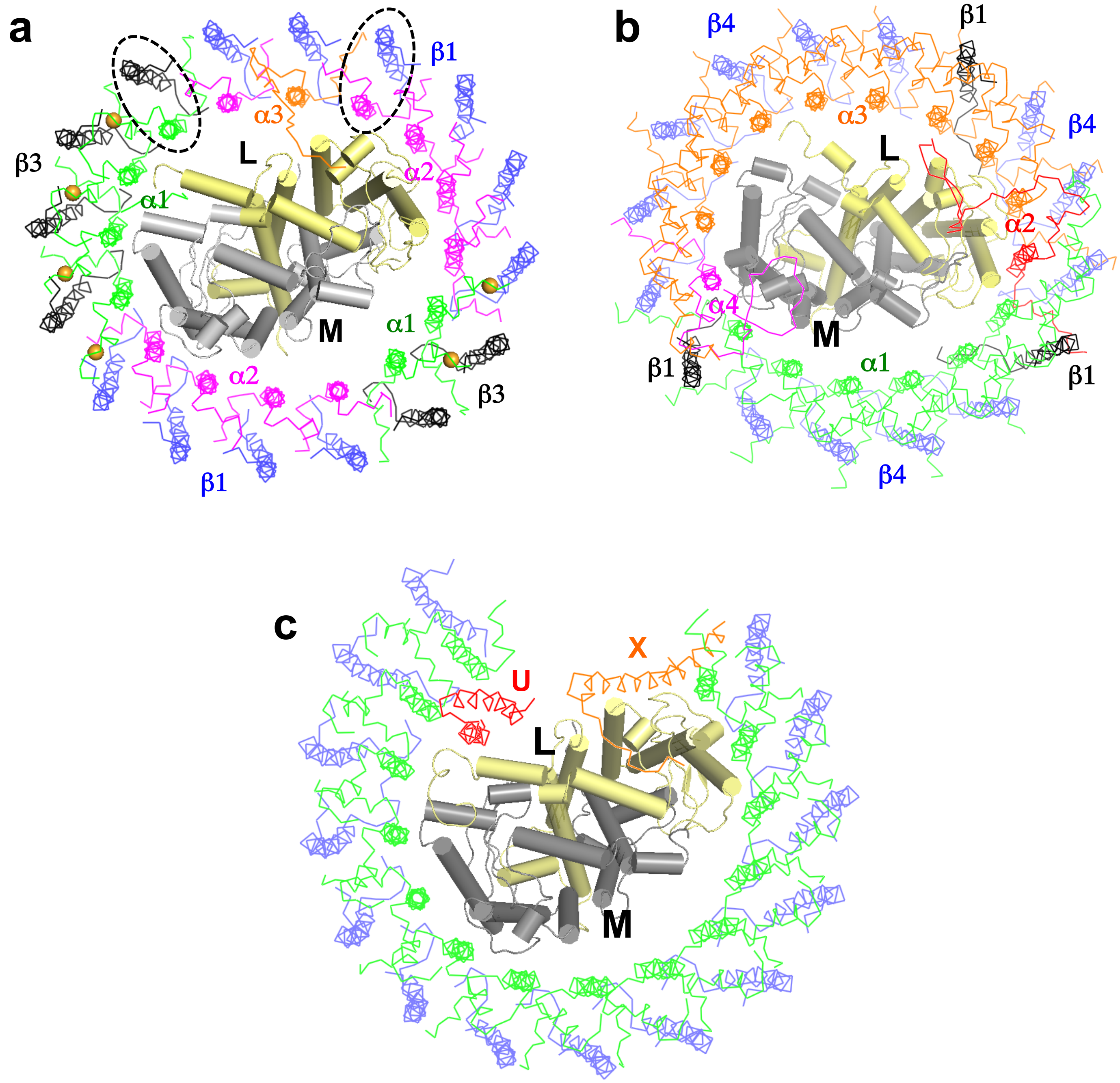

Figure S10. Comparison of arrangement of the *Alc. tepidum* LH1 αβ-polypeptides (a) with those of *Trv.* strain 970 (b) and *Rba. sphaeroides* strain IL106 (c). (a) Arrangement of the multiple *Alc. tepidum* LH1 αβ-polypeptides around the RC. Dashed ellipses indicate face-to-face dimeric subunits. **(b)** Arrangement of the multiple *Trv.* strain 970 LH1 αβ-polypeptides around the RC. **(c)** Arrangement of the LH1 αβ-polypeptides from *Rba. sphaeroides* strain IL106 around the RC, PufX (orange) and protein-U (red).

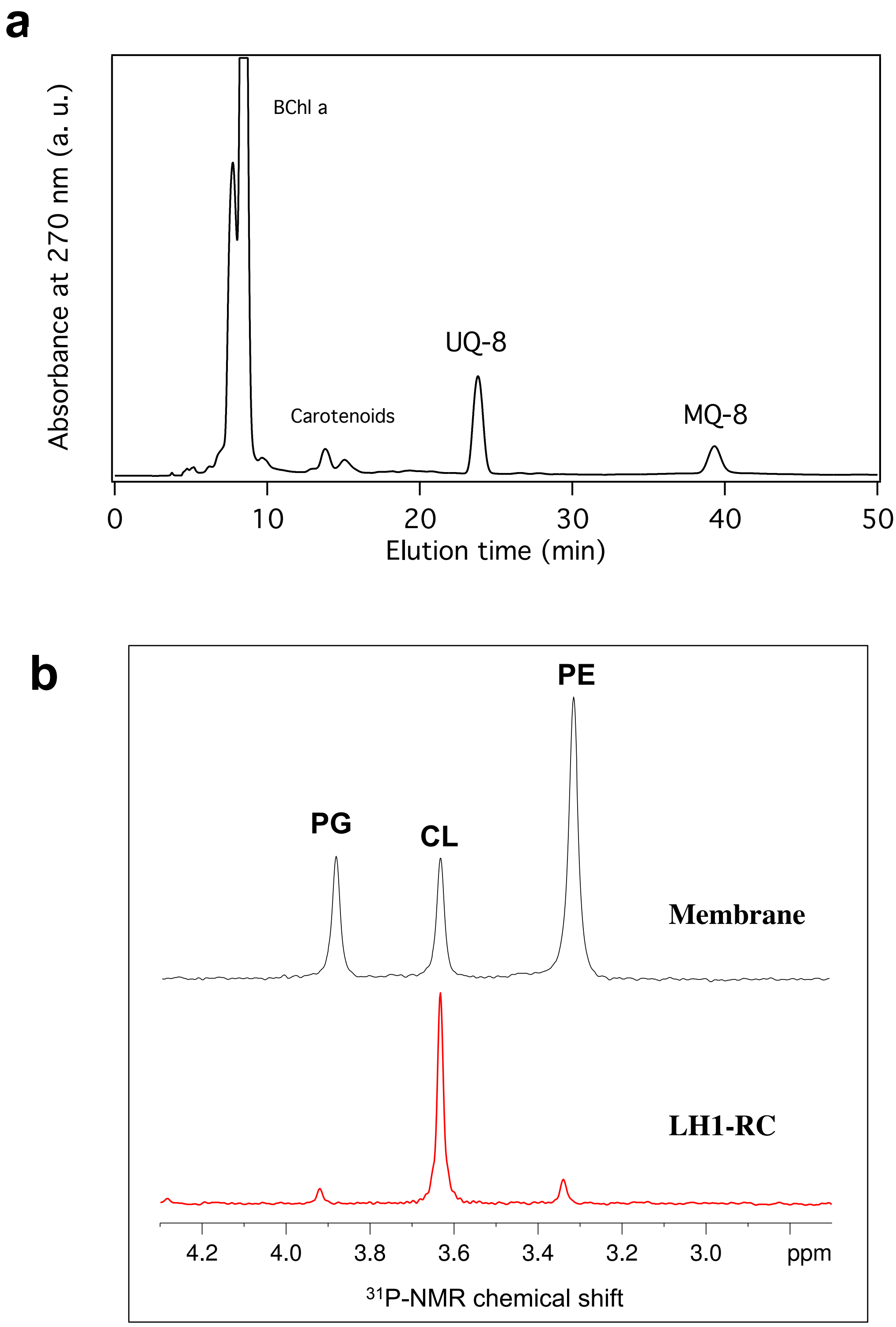

Figure S11. Identifications of the quinone molecules and phospholipid in *Alc. tepidum*. **(a)** Reverse-phase HPLC chromatograms (TSKgel, SuperODS, 4.6×100 mm, TOSOH) of the quinones and pigments extracted from the purified LH1-RC complex (25 ˚C, 7:3 methanol/isopropanol, flow rate 0.7 mL/min). **(b)** ^{31}P-NMR spectra of phospholipids extracted from the membrane and purified LH1-RC under the same conditions as described in the Materials and Methods section.
